# Green Grape Detection and Picking-Point Calculation in a Night-Time Natural Environment Using a Charge-Coupled Device (CCD) Vision Sensor with Artificial Illumination

**DOI:** 10.3390/s18040969

**Published:** 2018-03-25

**Authors:** Juntao Xiong, Zhen Liu, Rui Lin, Rongbin Bu, Zhiliang He, Zhengang Yang, Cuixiao Liang

**Affiliations:** College of Mathematics and Informatics, South China Agricultural University, Guangzhou 510642, China; liuz@stu.scau.edu.cn (Z.L.); limyui@stu.scau.edu.cn (R.L.); bobby@stu.scau.edu.cn (R.B.); hezhiliang@stu.scau.edu.cn (Z.H.); cxlyz@stu.scau.edu.cn (C.L.)

**Keywords:** green grapes, night-time environment, vision-sensor detection, picking-point calculation

## Abstract

Night-time fruit-picking technology is important to picking robots. This paper proposes a method of night-time detection and picking-point positioning for green grape-picking robots to solve the difficult problem of green grape detection and picking in night-time conditions with artificial lighting systems. Taking a representative green grape named *Centennial Seedless* as the research object, daytime and night-time grape images were captured by a custom-designed visual system. Detection was conducted employing the following steps: (1) The RGB (red, green and blue). Color model was determined for night-time green grape detection through analysis of color features of grape images under daytime natural light and night-time artificial lighting. The R component of the RGB color model was rotated and the image resolution was compressed; (2) The improved Chan–Vese (C–V) level set model and morphological processing method were used to remove the background of the image, leaving out the grape fruit; (3) Based on the character of grape vertical suspension, combining the principle of the minimum circumscribed rectangle of fruit and the Hough straight line detection method, straight-line fitting for the fruit stem was conducted and the picking point was calculated using the stem with an angle of fitting line and vertical line less than 15°. The visual detection experiment results showed that the accuracy of grape fruit detection was 91.67% and the average running time of the proposed algorithm was 0.46 s. The picking-point calculation experiment results showed that the highest accuracy for the picking-point calculation was 92.5%, while the lowest was 80%. The results demonstrate that the proposed method of night-time green grape detection and picking-point calculation can provide technical support to the grape-picking robots.

## 1. Introduction

With the rapid development of computer and automatic control technology, picking robots for fruits and vegetables have been gradually applied in agricultural production [1,2]. Grapes are a popular fruit and are produced on a large scale across the world. However, grape harvesting is time-consuming as well as labor-intensive. Therefore, by designing a grape-picking robot to realize automatic harvesting in a grape orchard this can raise grape production and increase income. This is of great importance for implementing automatic production in agriculture using picking robots.

There exists extensive research on the visual technology of picking robots so far, much of which focuses on visual recognition and location under natural light [3,4]. Bulanon et al. used luminance and the color difference of red (LCD) model to conduct segmentation of the Fuji apple using the optimal threshold with an accuracy of 88%. However, the error rate reached 18% in backlight circumstances [5]. Ji et al. conducted research on an apple-picking robot with a color charge-coupled device (CCD) camera installed on the end-effector to perform apple recognition. The picking experiments indoors and outdoors demonstrated that the success rate of apple recognition and positioning was 77% [6,7]. Chen et al. presented a vision-cognition framework for tomato harvesting which used the fusion of calibrated observation data from red, green and blue–depth sensors (RGB-D) installed on the head and the hand of a humanoid [8]. Bargoti and Underwood presented an image-processing framework based on multi-scale multi-layered perceptrons (MLP) and convolutional neural networks (CNN) for apple detection and counting using orchard image data. The research showed that using CNN and watershed segmentation (WS) resulted in the best performance for this dataset [9]. Chaivivatrakul and Dailey proposed a technique based on texture analysis, interest-point feature extraction and descriptor computation by using low-cost web camera sensors. This method has a high accuracy, with single-image detection rates of 85% for pineapples and 100% for bitter melons [10]. Kitamura and Oka designed a lighting system for the picking robot, which contained an image-processing system, a camera-positioning system, and a cutting device to improve the ability to recognize sweet peppers [11]. Font et al. developed an automatic fruit-harvesting system by using a stereovision camera to estimate the size, distance and position of the fruits. The robotic arm was used to mechanically pick up the fruits. The average distance error was from 4–5%, and the average diameter error was up to 30% in the case of a small object and in a range from 2–6% in the case of a pear and an apple [12]. Kusumam et al. used a low-cost RGB-D sensor under real-world conditions to address the tasks of detecting mature broccoli heads in the field and providing their 3D locations relative to the robotic vehicle [13]. Mehta et al. presented an estimation-based localization approach based on a new sensing procedure that uses multiple (≥2) inexpensive monocular cameras to estimate the unknown position of the fruits [14]. Rakun et al. described a method for apple fruit detection that relied on the combination of the object’s color, texture and 3D properties. This work could measure their size and model by estimating the fruit yield [15]. Sa et al. presented a novel approach for fruit detection of an autonomous agricultural robotic platform using deep CNN. They used color RGB and near-infrared (NIR) modalities for sweet pepper detection and the precision and recall performances improved from 0.807 to 0.838 [16]. Henten et al. focused on the individual hardware and software components of the robot, including the autonomous vehicle, the manipulator, the end-effector, the two computer-vision systems and a control scheme. With a success rate of 80%, the robot can pick cucumbers without human interference [17]. Stein et al. presented a novel multi-sensor framework that used a state-of-the-art faster regions with CNN features (faster R-CNN) detector. The pair-wise correspondences were established between images using trajectory data provided by a navigation system. In addition, a novel light-detection and ranging (LiDAR) component generates image masks for each canopy automatically, allowing each fruit to be associated with the corresponding tree. The experiment showed that single, dual and multi-view methods can all provide precise yield estimates, but only the proposed multi-view approach can do so without calibration, with an error rate of only 1.36% for individual trees [18]. Amatya et al. developed a machine-vision system for splitting and detecting cherry tree branches. For this system, a Bayesian classifier was used to segment the image, and then the curve-fitting method was used to connect the branches. The overall accuracy in detecting individual branches was 89.2% [19]. Yamamoto et al. developed a strawberry-harvesting robot and set up a visual system to detect strawberries in which white, red and green light-emitting diodes (LEDs) were used to increase detection accuracy, with a strawberry pick rate of 67.1% [20]. Among this research, mutable light conditions in natural environments are the key factors leading to recognition and positioning errors.

To solve the disturbance of changing natural light to picking robots, some researchers have performed visual recognition in night-time or greenhouse environments. Payne et al. developed the automatically estimated mango crop yield algorithm at night by reducing its dependence on color features and increasing its use of texture filtering and hessian filtering in particular [21]. Arefi et al. used a vision sensor and designed a recognition algorithm which could be adapted to the illumination conditions of a greenhouse. The total accuracy of the proposed algorithm was 96.36% [22]. Linker and Kelman developed a novel approach which was based on analyzing the spatial distribution of the light around highlights (“bright spots”) in night-time images under artificial illumination [23]. Qureshi et al. used texture-based dense segmentation as well as the shape-based fruit detection method for the automated counting of fruit in mango tree canopies by using night-time imaging [24]. A conclusion can be drawn from the above research that night-time visual technology for fruit detection or picking with stable artificial illumination can effectively improve the accuracy of fruit recognition and picking localization, avoiding the influence of different intensity or non-uniform distribution of natural light.

Currently, there exists some research on the visual recognition of grapes and several works mainly focus on recognition and positioning under natural light [25,26]. Dolezel et al. introduced and evaluated a classifier based on an artificial neural network for white wine grape recognition in a natural environment [27]. Since the natural light can influence the recognition performance, some research about night-time grape recognition has been undertaken. For instance, Reis et al. proposed a system for the detection and location in the night-time natural environment of bunches of grapes in color images. The system could distinguish white and red grapes and calculate the location of the bunch stem with recognition accuracies of 97% for red grapes and 91% for white grapes [28]. The above research mainly focuses on the recognition of grapes with different colors in different environments. The calculation of picking points is seldom discussed by researchers and also the speed of the algorithm of the picking robot is seldom considered.

The grape is a kind of multi-fruit, and its visual detection and location are more difficult than for a single-fruit species. Also, it is more difficult to recognize green grapes than red and brown ones in a natural environment. At the same time, natural lighting conditions can be a disturbance. Precise visual location is difficult for the grape picking robot. In this research, a kind of green grape named *Centennial Seedless* is selected to conduct the detection and picking-point calculation experiment at night-time. The Chan–Vese (C–V) level-set model proposed by both Chan and Vese is combined with the level-set idea and M-S mode [29,30]. In this paper, an algorithm based on the improved C–V level-set model was proposed to conduct night-time green grape detection. Combining the principle of the minimum circumscribed rectangle of fruit and the method of Hough straight-line detection, the picking point of the fruit stem was calculated. This proposed method of night-time green grape detection and picking-point calculation provides technical support to the grape-picking robots.

## 2. Materials and Methods

### 2.1. Image Acquisition

**Figure 1:**
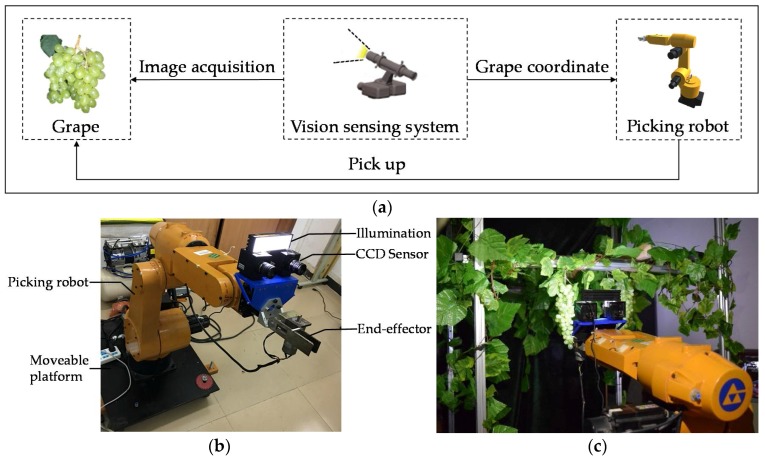
The picking robot and its vision-sensing system. (**a**) Vision-sensing process of the picking robot; (**b**) structure of the picking robot; (**c**) grape-picking experiment of the picking robot.

The picking robot and its vision-sensing system are shown in Figure 1. The vision-sensing system in this research consisted of two CCD cameras, LED illumination and the sensing algorithm. The CCD cameras used in the system were the MV-E800C produced by Microvision Corporation (Guangzhou, China), with a maximum resolution of 3312 × 2496 pixels. The illumination used consisted of the XREWHT-L1-Q5 LED lights produced by the CREE Corporation (Durham, NC, USA), with light temperature of 5700–7000 K, luminous flux of 93.9–100 LM·W^−1^, and a field angle of 90°, which can provide efficient stable illumination in a night-time environment. The structure of the grape-picking robot is shown in Figure 1b and the picking experiment of the night-time grape is shown in Figure 1c.

**Figure 2:**
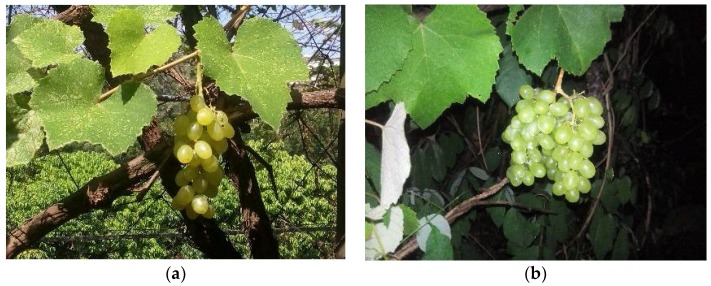
Daytime and night-time grape pictures. (**a**) Daytime grape cluster; (**b**) night-time grape cluster.

The lighting source was 50–100 cm from the grape fruit, in the same horizon level of the camera, parallel to the shooting direction. The acquisition time of the images was on 30 September 2016. The grape variety used was *Centennial Seedless*. The grape cluster was conical with an average weight of 1300 g and a maximum weight of 2500 g. Its fruit grain is elongated oval, close against, not easy to fall, of average weight 5.2 g and maximum weight 10 g. There were 324 daytime images and 637 night-time images in total. The resolution of the images was 3000 × 2000 pixels. The daytime images were collected with natural light, while the night-time images were collected with LED illumination. Examples of the daytime and night-time image are shown as Figure 2.

### 2.2. Color Feature Analysis of Images

**Figure 3:**
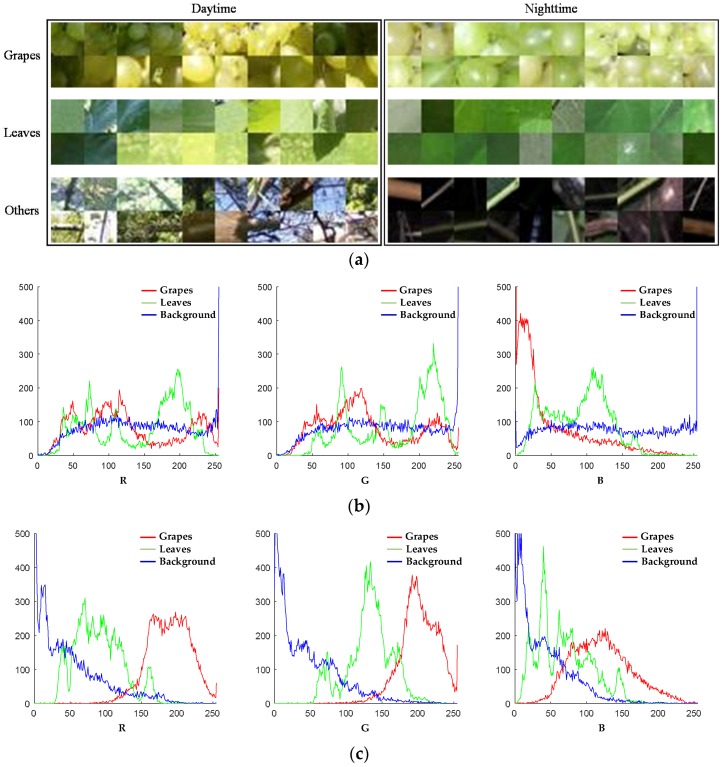
Color feature analysis of the acquired images. (**a**) Samples of image blocks; (**b**) color distribution of daytime red, green and blue (RGB) image; (**c**) color distribution of night-time RGB image.

The RGB color model as selected for the detection. We chose 50 image blocks of grapes, leaves and other parts, respectively, at daytime and night-time. There were 300 image blocks in total and the size of the blocks was 40 × 40. Samples of the image blocks are shown in Figure 3a. The blocks at daytime contained different lighting conditions like front lighting, back lighting and shading. The blocks at night-time were illuminated by artificial LED lighting. The histograms derived from these 300 blocks are shown in Figure 3b,c. With the lighting condition changing at daytime, the daytime images changed more in terms of brightness. Since the daytime histograms contained blocks of different lighting conditions, there were large amount of overlaps between the grape fruit and the background (leaf and other objects) in all three components of the RGB color model, while at night-time, the stable artificial illumination seldom changed the image brightness. This means that by controlling the lighting intensity, the grape could be recognized better. According to Figure 3c, the R component had least overlap between fruit and the background. In this component, the total brightness of the fruit was higher than the background. The brightness of the fruit was between 150 and 250, while the background was mainly lower than 150 and only a few parts were higher than 150. This indicated that the R component was suitable for the night-time detection of *Centennial Seedless*.

## 3. Grape Detection and Picking-Point Calculation

### 3.1. Algorithm Flow Diagram

**Figure 4:**
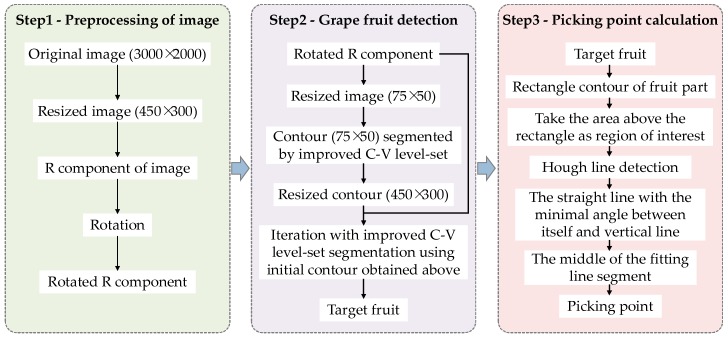
Algorithm flow diagram of the proposed method.

The proposed algorithm flow of this study is shown in Figure 4.

### 3.2. Image Pre-Processing

**Figure 5:**
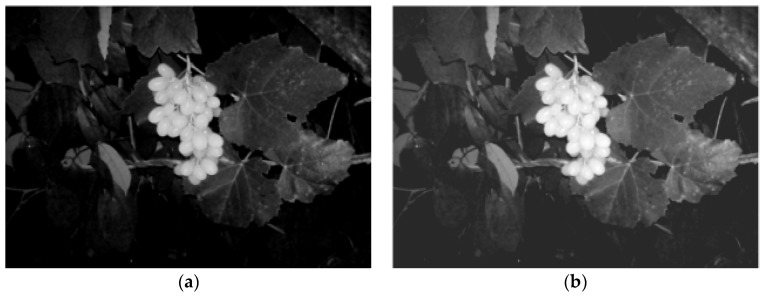

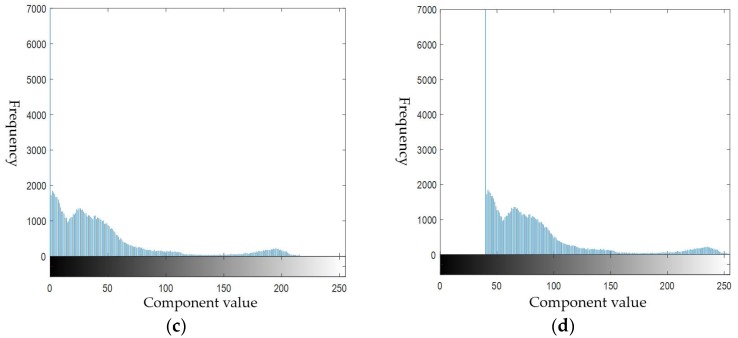
Comparison of R component before and after rotation. (**a**) The original R component image; (**b**) the R component image after rotation; (**c**) the original R component histogram; (**d**) the R component histogram after rotation.

In order to facilitate image batch processing, the resolution of the images was firstly adjusted to 300 × 450 pixels, and the R component of the RGB color model was extracted. Mao et al. used the rotation of Hue to move the high score range to the low point range, eliminating the reflective parts in some images, thus improving the recognition effect [31,32]. As shown in Figure 3c, under a stable artificial light environment, the R component of the grape area was mainly concentrated from 160 to 215. So, this paper according to Formula (1) rotated the R component values, and the treatment effect is shown in Figure 5. Through the use of the rotation of the R component, the grape component values move into the high-value range, the original component value is bigger than the grape component values and move to the low score interval, and the grape area become the largest area in the full component values. After the rotation of the R component, the background area of the high score value caused by the reflection, the external light source or other factors was moved to the low value range. Therefore, the component value of the background region was in the low value range, and the component value of the grape region was in the high score range, making the segmentation simpler and more precise.

(1) $$\left\{ \begin{array}{l} r_{1}=r+35,r+35\leq 255\\ r_{1}=r+35-255,r+35>255 \end{array}\right.,$$

where *r* is the component value of the original R component image, and $r_{1}$ is the component value of R component image after rotation.

### 3.3. Fruit Area Extraction

#### 3.3.1. Improved Chan–Vese (C–V) Level-Set Model

The gradient information was not utilized by the C–V level-set model, which evolves the curve by minimizing the energy function. An image *I*(*x*, *y*) with a definition domain of *Ω* is divided into two regions $C_{o}\;\mathrm{and}\;C_{b}$ of the target and background by a closed boundary, and their average grayscale value are $C_{o}\;\mathrm{and}\;C_{b}\;\mathrm{respectively}$. Then, the energy function is defined as follows:

(2) $$E(C,C_{o},C_{b})=\mu L(C)+vS_{o}(C)+\lambda_{o}{\int_{C_{o}}\left|I-C_{o}\right|}^{2}dxdy+\lambda_{b}{\int_{C_{b}}\left|I-C_{b}\right|}^{2}dxdy,$$

where L(C) is the length of the closed contour line *C*; and $S_{o}\left(C\right)$ is the internal region of $C_{o}$, namely the area of $C_{o}$, $\mu ,v\geq 0,\;\lambda_{0},\lambda_{b}\geq 0$ are the weight coefficients of the energy terms. The value of the energy function decreased as the iteration times inceeased. When E(C) reached the minimum, the contour curves of the two regions could be obtained. Using the traditional C–V level-set model, the level-set function needs to be updated within its definition domain with a large amount of calculation and long iteration time. Therefore, in this paper, based on method of other researchers [33,34], the image segmentation algorithm of the C–V level-set model is improved with better expression of the initialized level-set function. After the improvement, *C* will be shown as a straight line in plane Ω which is divided into an upper region $\Omega_{u}(y\geq y_{0})$ and a lower region $\Omega_{d}(y<y_{0})$, and the improved initialized function $\phi_{0}$ is shown as Formula (3):

(3) $$\phi_{0}\left(x,y\right)=\{\begin{array}{ll} \rho_{u}, & \left(x,y\right)\in \Omega_{u}\\ \rho_{d}, & \left(x,y\right)\in \Omega_{d} \end{array},\;\rho_{u}\times \rho_{d}<0,$$

where $\rho_{u}$ and $\rho_{d}$ are the fixed constants. Suppose $y=y_{0}$ to be the boundary between the upper and lower regions, the I (x,y) is processed using dispersed network segmentation with $y_{0}=j_{0}h$, and the Formula for calculation of $\phi^{n+1}$ can be evolved as:

(4) $$\frac{\phi_{i,j}^{n+1}-\phi_{i,j}^{n}}{\Delta t}=\{\begin{array}{c} \delta_{h}\left(\phi_{i,j}^{n}\right)R,\;j<j_{0}-1,j>j_{0}\\ \delta_{h}\left(\phi_{i,j}^{n}\right)\left(-\frac{u}{h}sign\left(\rho_{u}-\rho_{d}\right)+R\right),\;j=j_{0}\\ \delta_{h}\left(\phi_{i,j}^{n}\right)\left(\frac{u}{h}sign\left(\rho_{u}-\rho_{d}\right)+R\right),\;j=j_{0}-1 \end{array},$$

where δ(·) is regularization form of the Dirac function, $\phi_{i,j}^{n}=\phi \left(n\Delta t,x_{i},y_{j}\right)$; Δt is time step; h is the spatial step size of the discrete grid; i,j is the grid point coordinate; and u≥0 is an invariant parameter.

**Figure 6:**
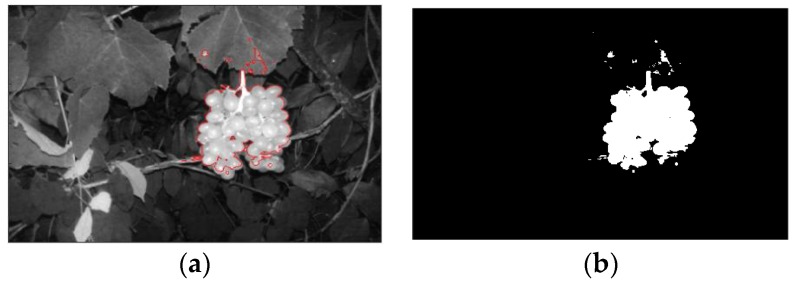
Segmentation with improved Chan–Vese (C–V) level-set model. (**a**) Segmentation result; (**b**) binary image.

The improved C–V level-set model improves efficiency of the horizontal set image segmentation from the aspects of time step and initialized curved surface. The level set of the improved model does not need to be reinitialized, the evolution time and the number of iterations are greatly reduced, and the segmentation efficiency is greatly improved with a better segmentation effect than traditional level-set model. The example of the segmentation effect using the improved C–V level set model is shown as Figure 6.

#### 3.3.2. Improved Algorithm Speed

The traditional C–V level-set model is applied with a large amount of computation and long operation time. It usually takes a few minutes to obtain results with the traditional model. The improved C–V level-set model increases the efficiency of the image segmentation. However, through testing, it is found that the running time of the algorithm using a R component image with 300 × 450 pixels is lengthy and not suitable for robot-picking jobs. Therefore, we propose an algorithm based on the improved C–V level-set model. Adopting the idea of the pyramid theory “Coarse to fine” [35], the resolution of the R component image is changed firstly to 50 × 75 pixels, and the grape fruit contour is obtained with the improved C–V level-set model; then the contour is enlarged to 300 × 450 pixels to obtain the rough contour. Using the enlarged contour as the initial contour of the level set, this continues to be iterated with the improved C–V level set to obtain a more accurate contour.

#### 3.3.3. Fruit Detection

The pseudo code of the image segmentation and target extraction was as follows (in Algorithm 1).

**Algorithm 1. The pseudo code of image segmentation and targets extraction****Input:** Night-time grape imageChange the image size to 300 × 450 pixels.Extract the R component histogram of the image.Calculate the number *a* of pixels with values greater than 140 and the number *b* of all pixels in the image.**If** (*a*/*b* > 7.75%).Rotate the R component image.Change the image size to 50 × 75 pixels and obtain the grape fruit contour using the improved C–V level set.Enlarge the grape fruit contour to 300 × 450 pixels as the initial contour, and iterate with the improved C–V level set to obtain a more accurate contour.Obtain binary image using the accurate contour.**For** all pixel regions (value for 1) in the binary image.Retain the largest region and fill the hole for every pixel region.Perform open and close operator to delete the noise.**End for.****End if.****Output:** Binary image that contains only the grape clusters.

There are four situations for grape images captured by the picking robot: (1) multi-fruit clusters in the same image; (2) fruit clusters out of the center light source; (3) no fruit cluster in the image; and (4) a stem connecting to the fruit is blocked. Since situation (4) is a complex comprehensive problem, it will not be considered in this paper.

**Figure 7:**
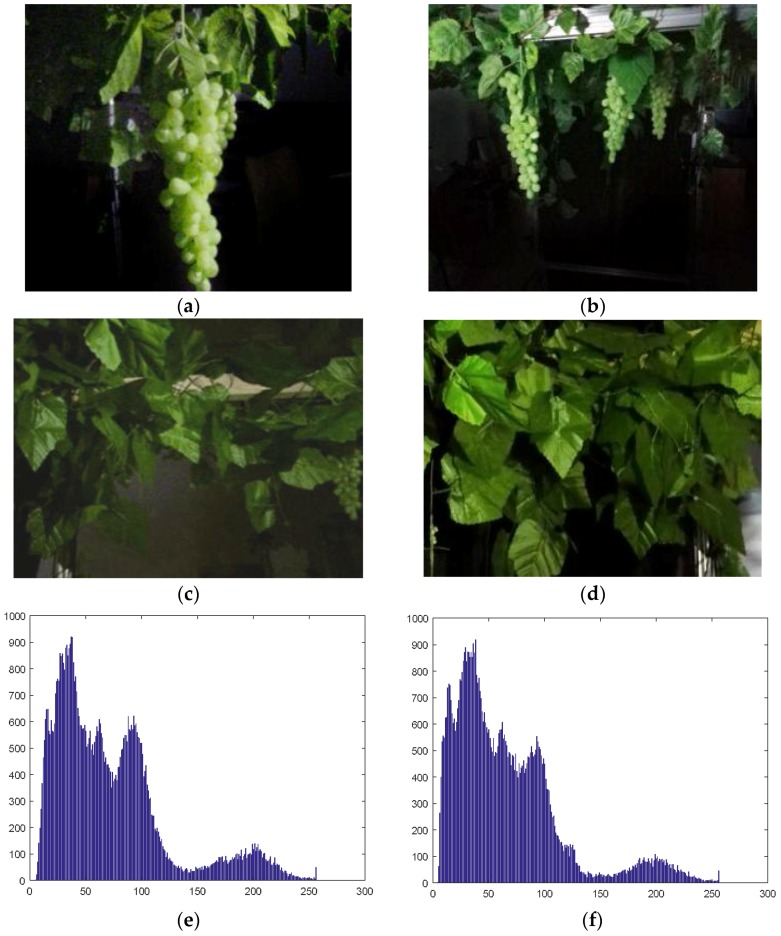

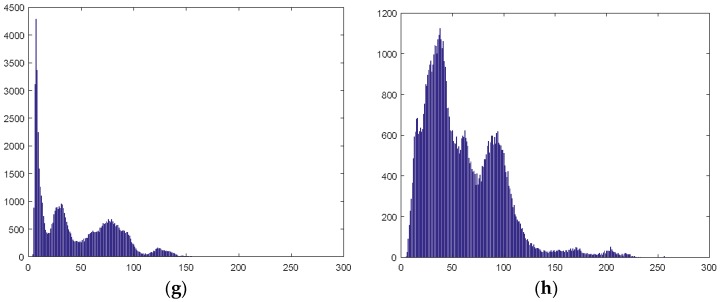
Histograms of the R component of the night-time images in different conditions. (**a**,**e**) Grape clusters near the light-source center; (**b**,**f**) grape clusters near light-source center and on the edge of the image; (**c**,**g**) grape clusters out of the light-source center and on the edge of the image; (**d**,**h**) no grape cluster.

For the first situation, multi-fruit clusters exist in the segmented binary image, and the largest fruit area will be reserved for further detection. For situations (2) and (3), according to the analysis results in Figure 7, there are some differences in the histograms for these different situations. There are mainly four types of images for discussion in the different histograms: (i) grape clusters near a light-source center; (ii) grape clusters near a light-source center and on the edge of the image; (iii) grape clusters on the edge of the image but not near a light-source center; and (iv) no grape cluster, shown in Figure 7.

The four types of images and their corresponding histograms are shown in Figure 7, from which we find the ratios of the R component values, which are greater than 140, and all values are different. Based on this feature, supposing the number of pixels with R component values over 140 to be *a* and the total pixel number to be *b*, 25 images of each type are chosen for analysis. In 50 images of the first two cases (as shown in Figure 7a,b), there are 49 images with the ratio of *a* and *b* greater than 7.75%, but the latter two cases (shown in Figure 7c,d) have a ratio of *a* and *b* less than 7.75%. So, we use Formula (5) to distinguish between the first two cases and the latter two. An image meeting Formula (5) can be proven to exist in a grape cluster in a light-source center or multi-grape clusters in a light-source center. If the image is not meeting Formula (5), it will not be further processed with detection for no grape cluster near a light-source center.

(5) $$\frac{a}{b}>7.75\%$$

In order to test the classification accuracy of Formula (5), 400 images were selected for testing, and the confusion matrix of test results was shown in Table 1. According to the test results of Table 1, 384 images of the 400 images were classified correctly and the classification accuracy was 96.0%.

Summarizing the aforementioned analyses, this research proposed a method to remove the background with procedures shown as follows:

Step 1. Extract the R component histogram of the night-time image. Then, calculate the number *a* of pixels with values greater than 140 and the number *b* of all pixels in the image. If Formula (5) is workable, go to Step 2. Otherwise, stop the grape picking.
Step 2. Perform segmentation of the grape image combining the improved C–V level-set segmentation. Then, conduct morphology processing and remove the minor noise.
Step 3. Calculate the number of connected regions. If the number is greater than 1, retain the largest region. If the number is equal to 1, the image is regarded as being a single grape cluster.

**Figure 8:**
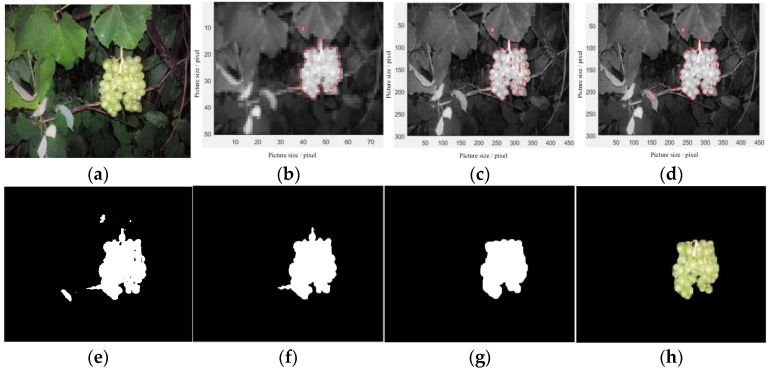
Detection results of the improved algorithm. (**a**) Night-time image; (**b**) initial iteration result; (**c**) rough contour; (**d**) further iteration result; (**e**) binary image; (**f**) Bbinary image after retaining the largest region and filling holes; (**g**) binary image after opening operation and closing operation; (**h**) grape fruit.

The improved C–V level-set was used to remove the background. Compared to the frequently changing lighting environment of daytime, the nocturnal image-capturing system has stable illumination, so segmentation of the grape image using the improved C–V level set is automatically stable. The segmentation effect of the proposed algorithm is shown as Figure 8.

### 3.4. Picking-Point Calculation

**Figure 9:**
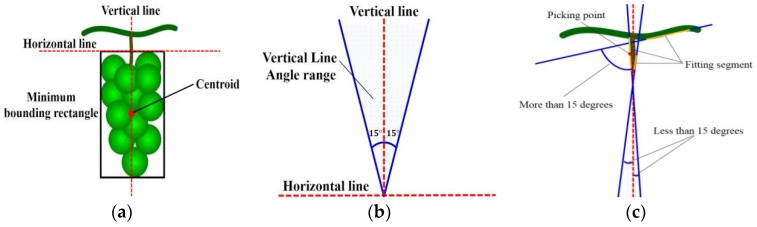

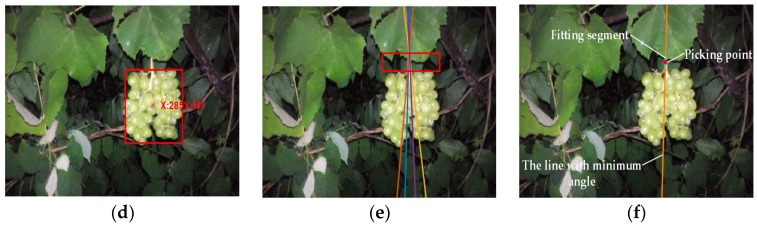
The calculation of the picking point. (**a**) Horizontal line and vertical line; (**b**) detection of line angle range; (**c**) determination of picking point; (**d**) fruit area; (**e**) the region of interest (red box) and the Hough detection results; (**f**) picking-point calculation.

The grape cluster is generally grown vertically to be under the action of gravity [36]. Based on this growth characteristic, the method of grape picking point calculation is proposed as follows:

Step 1. Determine the minimum enclosing rectangle of the grape fruits and figure out its centroid.
Step 2. Draw a vertical line through the centroid of the grape cluster, set the area above the rectangle to be the region of interest, detect straight lines of the region of interest using the Hough straight line-detection method (as the red box in Figure 9e), and remove straight lines with an angle between them and vertical lines greater than 15°, shown as Figure 9a,b.
Step 3. Calculate the angle of the remaining straight lines and the vertical line, take the fitting straight line with the minimal angle between itself and the vertical line as the line with the picking point, and take the middle point of the fitting segment as the picking point shown as Figure 9c.

The effect of using the above algorithm to calculate the grape picking point is shown as Figure 9d–f.

## 4. Results and Discussion

To validate the performance of the proposed algorithm, the experiments of green grape detection and picking-point calculation in a night-time environment were performed.

### 4.1. Grape Fruit Detection Experiment

This study designed detection experiments using 300 night-time images. We first resized these images into 300 × 450. Then we used Photoshop to label the fruit area artificially; this can provide criteria to evaluate the algorithm. Detection results were compared with the artificial labels. In order to facilitate the analysis of the experimental data, the percentage of correct area (PCA) and the percentage of false area (PFA) were used evaluate the results, as defined by Formulas (6) and (7):

(6) $$\mathrm{Percentage}\;\mathrm{of}\;\mathrm{correct}\;\mathrm{area}=\frac{\mathrm{grape}\;\mathrm{pixels}\;\mathrm{recognized}\;\mathrm{correctly}}{\mathrm{all}\;\mathrm{grape}\;\mathrm{pixels}}\times 100\%$$

(7) $$\mathrm{Percentage}\;\mathrm{of}\;\mathrm{false}\;\mathrm{area}=\frac{\mathrm{pixels}\;\mathrm{recognized}\;\mathrm{falsely}\;\mathrm{as}\;\mathrm{grape}}{\mathrm{all}\;\mathrm{pixels}\;\mathrm{recognized}\;\mathrm{as}\;\mathrm{grape}}\times 100\%$$

**Figure 10:**
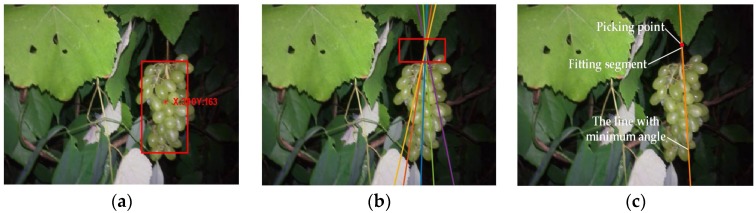

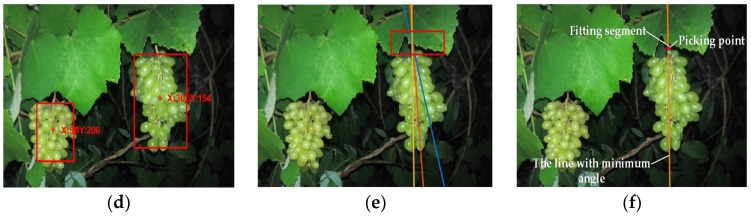
The successful detection results. (**a**,**d**) Fruit area; (**b**,**e**) the region of interest (red box) and the Hough detection; (**c**,**f**) picking-point calculation.

The detection results and picking-point calculations are shown in Figure 10. Table 2 shows the experimental results. For the PCA value, 275 images were over 90%; 12 images were between 70% and 90%; and the remaining 13 images were of lower accuracy. Regarding images over 90% as correct results, the accuracy rate for night-time grape detection was 91.67%. For the PFA value, 271 images were less than 5%; 16 images were between 5% and 15%; and the remaining 13 images were greater. Regarding images over 5% as wrong results, the error rate for night-time grape detection was 9.67%. Table 3 is the confusion matrix of grape fruit detection. The correct rate of fruit pixel detection is 91.70%, while it was 93.66% for the background pixels. The experimental results show that the proposed algorithm can detect the grape fruits effectively in the night-time natural environment and would be good guidance for the night-time operation of green grape-picking robots.

According to the experimental results above, in the daytime environment the color and texture feature of the fruit and the leaf change as the lighting condition does. The acquired images in this environment have a complex background containing earth, sky and buildings, while in the night-time environment, the intensity of the illumination is controllable. The influence of the surroundings can be reduced greatly, which can improve the correct rate of visual detection effectively.

**Figure 11:**
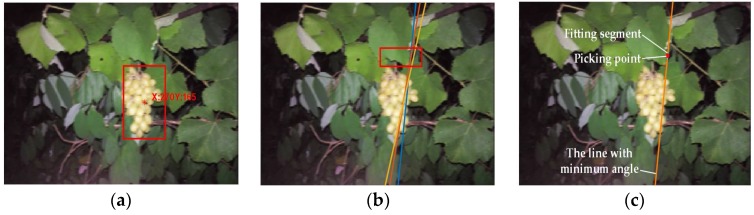

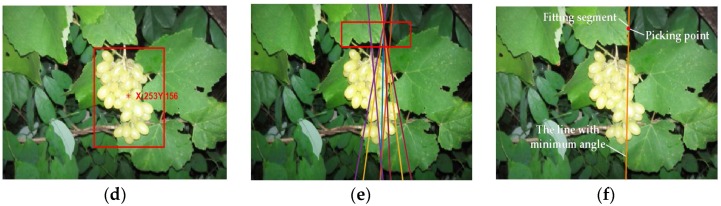
The error results of grape-detection and picking-point calculation. (**a**,**d**) Fruit area; (**b**,**e**) the region of interest (red box) and the Hough detection; (**c**,**f**) picking-point calculation.

The error results of grape detection and picking-point calculation are shown in Figure 11. There are two main reasons: (1) the captured grape image becomes blurred resulting in a detection error because of relative motion between the camera and target grape cluster. (2) Some grape-detection errors were caused by inappropriate control of the distance between the camera and target grape cluster. A light spot appears in the grape image under conditions of relatively short distance between the illumination equipment and the grape cluster, while dark images occur under condition of relatively long distance. In order to reduce the possible grape-detection errors, the following measures can be taken: (1) the distance between the illumination equipment and the target grape cluster should be controlled well. In addition, the distance-measuring device can be added to the image-capturing system and brightness of the LED lamp should be changed according to the measured data. (2) The image-processing algorithm should be optimized for grape clusters with a disturbance in our further study.

### 4.2. Picking-Point Calculation Experiment

The picking-point calculation experiment was carried out using the proposed method with 240 night-time green grape images. When the calculated picking point is on the stem of the grape cluster, or out of the stem but with a horizontal distance to the stem of less than or equal to 5 pixels, the picking-point calculation is considered to be successful. Then, night-time images were captured with a depth distance of 50 to 100 cm between the camera and the grapes. The distance was determined by a laser rangefinder. A picking-point calculation was conducted using the proposed algorithm, and the results are shown in Table 4.

A calculated picking point with a distance of fewer than 5 pixels to the horizontal bearing branch is marked as a successfully recognized picking point, while others are marked as erroneous picking points. The accuracy rate is the ratio of the successful calculation number and the total image number. There is a different accuracy rate of picking-point detection for images captured at different depth distances. The highest accuracy rate is 92.5% for a depth of 500 mm, while the lowest is 80.0% for a depth of 1000 mm. It can be seen from the experimental results that the proposed method effectively calculates the grape picking point in the night-time natural environment.

The error results of picking-point calculation are shown in Figure 11. The specific reasons for calculation errors of picking-point determination are found as follows: (1) grape fruit detection failure leads to picking-point calculation errors. (2) The light intensity decreases with increase of the shooting distance, by which the quality of night-time images is influenced, resulting in a picking-point calculation error. The following measures can be taken for avoiding picking-point calculation errors: (1) the image quality can be improved by optimizing the lighting system; (2) the grape fruit-detection algorithm can be improved to increase the accuracy of detection in further studies.

### 4.3. Algorithm Running Time Experiment

In order to evaluate the running time of the algorithm, this study designed a real-time experiment. MATLAB 2016a was used to test the given algorithm on a PC running 64-bit Windows 7 with 8 G RAM and 4 cores 3.4 GHz CPU. Fifty night-time images of grape fruit were used for recognition employing the proposed, traditional and improved algorithms. The average running time of the segmentation procedure was 135.4 s using the traditional C–V level-set model in segmenting 300 × 450 pixels images, while the average running time using the improved C–V level-set model was 11.8 s. Both the above algorithms cannot meet the requirement of robot-picking jobs. The idea of the pyramid theory “Coarse to fine” is used in our algorithm by the 50 × 75 R component image first being processed with the improved C–V level-set model, with its iterative result being amplified, second, to obtain a rough contour as the initial contour of the C–V level set, and finally iterative computation being continued with the improved C–V level set model to obtain the accurate contour. This algorithm can greatly shorten the average running time of grape detection while maintaining a good segmentation effect. The average running time of our algorithm was 0.46 s, satisfying the requirement of robot-picking jobs. From image input to result return (the coordinates of picking point or the cessation command), the average running time of the night-time images was 0.58 s.

## 5. Conclusions and Future Work

The accurate detection of grape fruits and the precise location of picking points are both critical to the successful operation of grape-picking robots. Green grape fruit detection and the picking-point calculation method were studied in this paper to avoid daytime grape-detection errors influenced by mutable natural light conditions. The effectiveness and feasibility of the proposed algorithm was proven by the experimental results.

(1) Color models of the night-time grape images are analyzed using the exploratory analysis method with a result that the R component of the RGB grape image is suitable for implementation of night-time image detection of the green grape cluster.
(2) Based on the R component of the night-time grape image, the background of the grape image is removed by the improved C–V level-set model combined with morphological treatment. The visual-detection experiment results show that the accuracy of grape fruit detection was 91.67% or more, and the average running time of the proposed algorithm was 0.46 s.
(3) According to the growth characteristics of the grapes, the Hough line detection method was used to fit the fruit stem above the fruit, and the picking points on the fruit stem were determined. The experimental result of picking-point calculation of the night-time grape cluster showed that the highest accuracy rate was 92.5% for a depth of 500 mm while the lowest was 80.0% for a depth of 1000 mm. From image input to result return (the coordinates of the picking point or the cessation command), the average running time of the night-time images was 0.58 s. This study provides technical support for grape-picking robots in a natural environment.

In conclusion, the given algorithm can make grape detection and picking-point calculation successful and effective. However, the overlap phenomenon and the moving disturbances have not been solved. Further study will be conducted and 3D modelling of the scene will be constructed to solve these problems in the future.

## Figures and Tables

**Table 1:** The confusion matrix of Formula (5) classification results.

Reality	Classification Results			
	*a*		*b*	
	Amounts	Ratio	Amounts	Ratio
*a* ^1^	193	96.5%	7	3.5%
*b* ^2^	9	4.5%	191	95.5%

^1^ Exists in a grape cluster in a light-source center or multi-grape clusters in a light-source center. ^2^ No grape cluster near a light-source center.

**Table 2:** Statistical results of grape-detection experiment.

		Percentage of Correct Area			Percentage of False Area		
		>90%	70~90%	<70%	<5%	5~15%	>15%
Total image	300	275	12	13	271	16	13
Ratio	100%	91.67%	4.00%	4.33%	90.33%	5.33%	4.33%

**Table 3:** Confusion matrix of segmentation result.

Reality	Segmentation Result			
	Fruit Pixels		Background Pixels	
	Amounts	Ratio	Amounts	Ratio
Fruit pixels	34,545,372	91.70%	3,125,679	8.30%
Background pixels	179,314	6.34%	2,649,635	93.66%

**Table 4:** Accuracy of picking-point calculation.

Depth/mm	Number of Images with Visible Grapes	Pixels Error In Row			Accuracy Rate/% ^1^
		0/Pixel	1~5/Pixel	>5/Pixel	
500	40	26	11	3	92.5
600	40	24	12	4	90.0
700	40	23	13	4	90.0
800	40	18	16	5	85.0
900	40	15	19	6	85.0
1000	40	10	22	8	80.0

^1^ A picking point with an error of fewer than 5 pixels (column 3 and 4) is regarded as a successfully recognized point.
